# KOunt: a reproducible KEGG orthologue abundance workflow

**DOI:** 10.1093/bioinformatics/btad483

**Published:** 2023-08-03

**Authors:** Jennifer Mattock, Marina Martínez-Álvaro, Matthew A Cleveland, Rainer Roehe, Mick Watson

**Affiliations:** The Roslin Institute and Royal (Dick) School of Veterinary Studies, University of Edinburgh, Edinburgh, Midlothian, United Kingdom; Scotland’s Rural College, Edinburgh, United Kingdom; Genus plc, DeForest, WI, United States; Scotland’s Rural College, Edinburgh, United Kingdom; Scotland’s Rural College, Edinburgh, United Kingdom; Centre for Digital Innovation, DSM Biotechnology Center, Delft, The Netherlands

## Abstract

**Summary:**

Accurate gene prediction is essential for successful metagenome analysis. We present KOunt, a Snakemake pipeline, that precisely quantifies KEGG orthologue abundance.

**Availability and implementation:**

KOunt is available on GitHub: https://github.com/WatsonLab/KOunt. The KOunt reference database is available on figshare: https://doi.org/10.6084/m9.figshare.21269715. Test data are available at https://doi.org/10.6084/m9.figshare.22250152 and version 1.2.0 of KOunt at https://doi.org/10.6084/m9.figshare.23607834.

## 1 Introduction

Accurate and effective sequence annotation is key in interpreting metagenomic sequence data. The KEGG database is a popular reference database that groups proteins into functional orthologs, termed KEGG orthologs (KOs) (Kanehisa *et al.* 2022). Several tools that identify KO abundance exist with varying aims. FMAP is a functional analysis pipeline that aligns reads to a KEGG filtered UniProt reference database and calculates gene family abundance (Kim *et al.* 2016). DiTing uses KofamKOALA to identify KOs and calculates relative abundance (Xue *et al.* 2021). Both HumanN2 and Metalaffa provide conversion between UniRef90 hits and KOs; HumanN2 also allows searching against a legacy version of the KEGG database (Franzosa *et al.* 2018, Eng *et al.* 2020).

Here, we describe KOunt, a reproducible workflow which uses freely available software to calculate KO abundance in metagenomic sequence data, taking multiple approaches to improve the annotation of proteins and reads that initially do not have a hit. Unlike other KO abundance tools, KOunt gives the user the option to calculate the abundance of the RNA KOs in the metagenomes and also cluster the proteins by sequence identity to report the diversity within each KO. KOunt has been used to successfully quantify KO abundance in rumen microbiome samples (Martínez-Álvaro *et al.* 2022).

## 2 Features

KOunt uses Snakemake to generate a scalable, reproducible workflow, utilizing freely available software (Köster and Rahmann 2012, Grüning *et al.* 2018). The pipeline is accompanied by reads subsampled from ERR2027889 to quickly test that installation has completed successfully. Reads are trimmed, assembled, proteins predicted, and coverage calculated with Fastp, Megahit, Prodigal, and BEDTools, respectively (Hyatt *et al.* 2010, Quinlan and Hall 2010, Li *et al.* 2015, Chen *et al.* 2018). Complete proteins are annotated with a KO using KofamScan and can be filtered by coverage evenness (Aramaki *et al.* 2020). These proteins are subsequently clustered by 100%, 90%, and 50% sequence identity with CD-Hit and MMseqs2 to quantify the diversity within each KO (Li and Godzik 2006, Steinegger and Söding 2017).

Users then have the option of using the custom KOunt databases to further annotate proteins and reads without a hit. Proteins and reads are aligned against the KOunt protein and RNA databases with Diamond and MMseqs2 and then assessed for RNA presence using kallisto (Bray *et al.* 2016, Buchfink *et al.* 2021). An in-depth description of the pipeline is available in Supplementary Information.

## 3 Results and discussion

**Figure 1.**
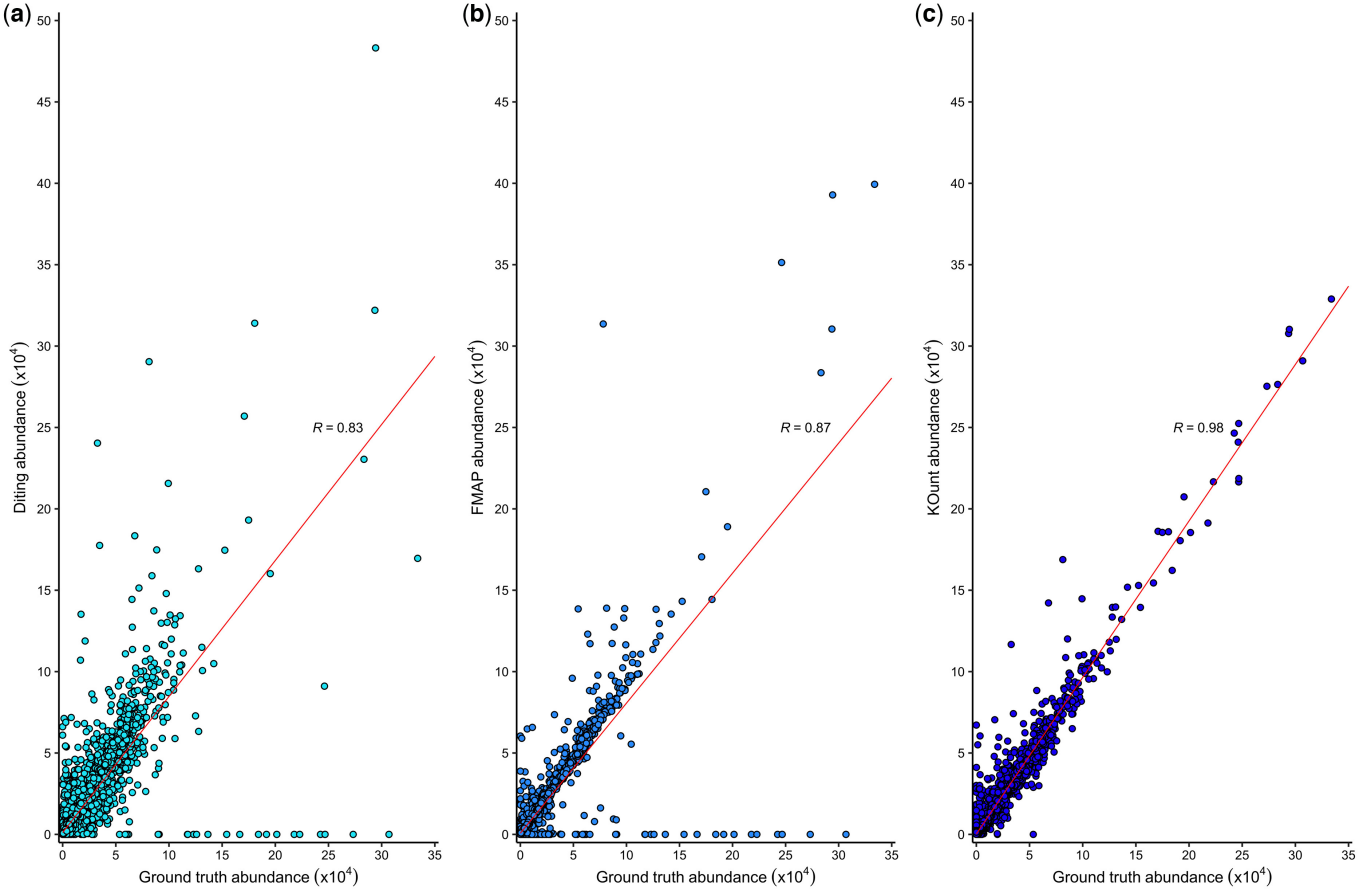
Comparison of ground truth KO abundance and DiTing, FMAP and KOunt KO abundance. (a) Ground truth versus DiTing KO abundance, (b) Ground truth versus FMAP KO abundance, (c) Ground truth versus KOunt KO abundance

To benchmark KOunt against other KO abundance software, we ran KOunt, FMAP, and DiTing with simulated metagenomic reads of organisms from the human and rumen gut microbiotas; the methods for this are available in Supplementary Information. Figure 1 illustrates the KO abundance, summed across the 10 samples, of the 3 approaches compared to the ground truth data. KOunt had the highest correlation with the ground truth data (*r* = 0.98 ± 0.0003) when compared with FMAP (*r* = 0.87 ± 0.002) and DiTing (*r* = 0.83 ± 0.003). DiTing both missed high abundance KOs and overestimated several, such as K07497 whose abundance increased from 294 342 in the ground truth results to 483 177. FMAP had a better correlation to the ground truth (*r* = 0.87 ± 0.002) but was still missing many high abundance KOs. KOunt was able to annotate the high-abundance KOs missed by the other approaches; many of these were RNA, which KOunt accurately quantified unlike DiTing and FMAP. When comparing only the KOs identified by all methods, KOunt was still more accurate (*r* = 0.98 ± 0.0004) than FMAP (*r* = 0.97 ± 0.0006) or DiTing (*r* = 0.92 ± 0.0017).

Of the 12 945 KOs present in the reads according to the KEGG annotation, KOunt found the most at 11 343, followed by FMAP with 10 735 and DiTing with 9681. Whilst KOunt performed the best at identifying KOs reported in the ground truth, it also found the largest number of KOs (1575) not reported by the ground truth, versus 1228 and 188 by FMAP and DiTing, respectively (Supplementary Figure S1). This could indicate that KOunt finds more false positives than the other approaches; however, we think it’s likely that, due to the multitude of approaches KOunt uses to quantify proteins, KOunt is identifying proteins that were not in the KEGG database when the genomes were originally annotated.

Many proteins from microbiomes cannot be annotated to a known protein sequence, for example 40% of the 170 million proteins in the Unified Human Gastrointestinal Genome collection are unannotated (Almeida *et al.*, 2021). Therefore, retaining as many reads as possible while maintaining accuracy is paramount. Across the 10 samples, FMAP and DiTing assigned on average 78 million and 79 million reads, respectively, to a KO; KOunt outperformed both, capturing an average of 116 million reads per sample. Whilst this is clearly beneficial, as 150 million reads are in the simulated datasets, there is still a need for improved protein annotation of reference datasets.

KOunt also clusters the proteins identified by KofamScan by sequence identity, allowing investigation of the diversity within KOs. In this dataset, without evenness filtering, 3 million proteins were identified by KofamScan, which grouped into 0.4 million 90% clusters and 0.2 million 50% clusters. K03406, methyl-accepting chemotaxis proteins, was the KO with the largest number of 50% clusters (1311) identified with KOunt, as a protein needs to have just 50% similarity to one of the proteins in a cluster to be included in that cluster, this illustrates the vast amount of diversity within this KO. The grouping of homologous proteins enables further investigation of highly abundant clusters and those with abundance associated with traits of interest.

To conclude, we present KOunt, a reproducible, scalable pipeline which accurately calculates raw KO abundance from metagenomic sequencing reads. Furthermore, KOunt also reports the number of 90% and 50% sequence identity clusters in each KO, showing the protein diversity within the KOs and facilitating exploration of groups of unannotated proteins.

## Supplementary Material

btad483_Supplementary_Data

## Data Availability

The KOunt pipeline is available at https://github.com/WatsonLab/KOunt. The KOunt reference database is available on figshare: https://doi.org/10.6084/m9.figshare.21269715. Test data are available at https://doi.org/10.6084/m9.figshare.22250152 and version 1.2.0 of KOunt at https://doi.org/10.6084/m9.figshare.23607834.
