# Supplementary figures and images for: KOunt: a reproducible KEGG orthologue abundance workflow
Source: Bioinformatics. 2023 Aug 3;39(8):btad483. doi: 10.1093/bioinformatics/btad483 (PMC10423021; doi:10.1093/bioinformatics/btad483)

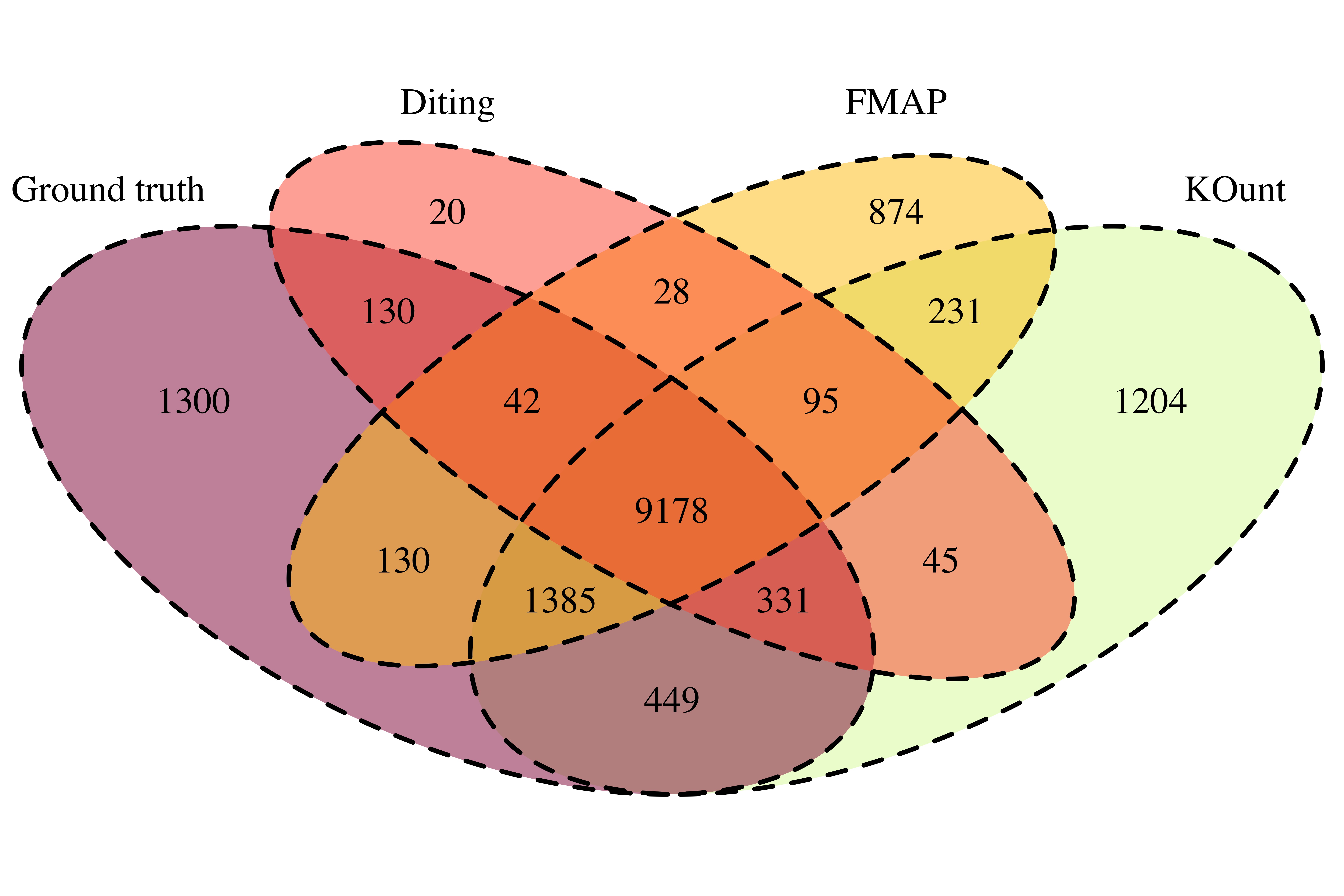

Supplement: btad483_Supplementary_Data [file btad483_supplementary_data.zip › Supplementary Figure 1.jpeg]
